# Immunotherapy and Targeted Therapy for Hepatocellular Carcinoma: A Literature Review and Treatment Perspectives

**DOI:** 10.3390/ph14010028

**Published:** 2020-12-31

**Authors:** Daniel M. Girardi, Jana Priscila M. Pacífico, Fernanda P. L. Guedes de Amorim, Gustavo dos Santos Fernandes, Marcela C. Teixeira, Allan A. L. Pereira

**Affiliations:** 1 Hospital Sírio-Libanes, SGAS 613/614 Conjunto E Lote 95-Asa Sul, Brasília 70200-730, Brazil; gustavo.hemato@gmail.com (G.d.S.F.); allan.pereira.onco@gmail.com (A.A.L.P.); 2 Hospital de Base do Distrito Federal, SMHS-Área Especial, Q. 101-Asa Sul, Brasília 70330-150, Brazil; marcelacrosara71@yahoo.com.br; 3 Escola Superior de Ciências em Saúde, SMHN Conjunto A Bloco 01 Edifício Fepecs-Asa Norte, Brasília 70710-907, Brazil; jana.priscila@hotmail.com (J.P.M.P.); felima_1611@yahoo.com.br (F.P.L.G.d.A.); 4 Hospital DF Star, SGAS I SGAS 914-Asa Sul, Brasília 70390-140, Brazil

**Keywords:** hepatocellular carcinoma, immune checkpoint inhibitors, targeted therapy, biomarkers

## Abstract

Advanced hepatocellular carcinoma is a prevalent and potentially aggressive disease. For more than a decade, treatment with sorafenib has been the only approved therapeutic approach. Moreover, no agent has been proven to prolong survival following the progression of disease after sorafenib treatment. However, in recent years, this scenario has changed substantially with several trials being conducted to examine the effects of immunotherapy and novel targeting agents. Several immune checkpoint inhibitors have shown promising results in early-stage clinical trials. Moreover, phase III trials with large cohorts have demonstrated remarkable improvement in survival with the use of new targeted therapies in second-line treatment. Treatment regimens involving the combination of two immune checkpoint inhibitors as well as immune checkpoint inhibitors and anti-angiogenic targeted therapies have shown potential to act synergistically in clinical trials. Recently, the combination of atezolizumab and bevacizumab evaluated in a phase III clinical trial has demonstrated survival superiority in the first-line treatment; it is the new considered standard of care. In this manuscript, we aimed to review the latest advances in the systemic treatment of advanced hepatocellular carcinoma focusing on immunotherapy and targeted therapies.

## 1. Introduction

Hepatocellular carcinoma (HCC) is a major health problem worldwide. It is estimated to be the sixth most common cancer, the fourth leading cause of cancer-related deaths [1], and the most common primary liver cancer, accounting for up to 90% of the cases [2]. HCC often originates in an inflamed cirrhotic liver, frequently due to chronic hepatitis B or C, chronic exposure to toxic agents (alcohol and aflatoxins), metabolic syndromes (non-alcoholic fatty liver disease and diabetes), and diseases associated with the immune system (primary biliary cirrhosis and autoimmune hepatitis) [2,3]. Despite the advances made in the development of approaches to the early detection of HCC, many patients are first diagnosed at an advanced stage [4,5].

Several staging systems have been proposed for clinical classification and prediction of survival. Among these, the Barcelona Clinic Liver Cancer (BCLC) staging system has been the most commonly used method to guide treatment decisions [6]. Liver function is also crucial for making treatment decisions and is usually assessed according to the Child–Turcotte–Pugh criteria, which evaluate the degree of ascites, concentrations of albumin and bilirubin in the serum, prothrombin time, and degree of encephalopathy. A scoring system is applied to each category and patients are classified into three groups that correlate with survival: Child–Pugh score of 5 to 6 is considered class A (well-compensated illness), 7 to 9 is class B (significant functional impairment), and 10 to 15 is class C (decompensated disease) [7,8].

For early-stage HCC (BCLC A), curative treatment includes liver transplantation, surgical resection, or radiofrequency ablation [9,10]. For intermediate-stage HCC (BCLC B), which presents a large or multifocal tumor mass without extrahepatic invasion, transarterial chemoembolization or selective internal radiation therapy is the recommended treatment [11].

For patients with advanced disease (BCLC C), treatment with sorafenib has been the standard of care for more than a decade based on the findings of two phase III trials (SHARP and Asia–Pacific) showing improved overall survival (OS) in patients who received sorafenib treatment. The SHARP trial randomly assigned 602 patients with advanced HCC, Child–Pugh liver function class A, who had not received previous systemic treatment to receive either sorafenib (400 mg twice daily) or placebo. Median OS was 10.7 months in the sorafenib group and 7.9 months in the placebo group (HR: 0.69; *p* < 0.001) [12]. The Asia–Pacific trial enrolled 226 patients with advanced HCC who had not received previous systemic therapy and had Child–Pugh liver function class A. Patients were randomly assigned to receive either oral sorafenib (400 mg) or placebo twice daily in 6-week cycles. Median OS was 6.5 months in patients treated with sorafenib, compared with 4.2 months in those who received placebo (HR: 0.68; *p* = 0.014) [13]. Fortunately, treatment options for patients with advanced HCC have recently improved with the approval of new targeted agents and immune checkpoint inhibitors (ICIs). This paper aims to review the latest new treatment options in first- and second-line therapies for HCC.

## 2. Checkpoint Inhibitors

Immune checkpoints are specific subtypes of membrane-bound molecules that act as pivotal regulators of immune escape in cancer [14]. The development of ICIs has been the most significant advance made in oncology in the last decade, and their use for the treatment of cancer patients has grown rapidly since 2011 [15]. Moreover, the percentage of patients who benefit from ICIs is also growing [15]. Cytotoxic T lymphocyte protein 4 (CTLA-4) and programmed cell death protein-1 and its ligand (PD-1, PD-L1) are some of the immune checkpoints targeted in HCC clinical trials [16]. These molecules act as negative regulators of T cell activation and their main function is to prevent autoimmunity by participating in the pathway to distinguish self- from non-self-antigens [17]. PD-1 participates in the inhibition of T cell activity in the effector phase, while CTLA-4 participates in the regulation of immune responses early in the T cell activation pathway. In cancer, these molecules are involved in the ability of tumor cells to escape the surveillance and elimination by the immune system; therefore, inhibition of these checkpoints is involved in the enhancement of anticancer immunity [18].

Previous studies have shown that the expression of PD-L1 increases during chronic viral infection and other inflammatory liver disorders, which leads to a higher tolerance toward tumor-associated antigens and favorable conditions for HCC tumorigenesis [19]. Chronic inflammation in the liver is associated with an augmented number of regulatory T cells as well as altered expression of checkpoint molecules and dendritic cell function, which inhibits an immune attack on the infected hepatocytes [20]. Moreover, increased expression and upregulation of PD-1 is associated with the progression of hepatic cirrhosis associated with hepatitis B virus (HBV) infection to HCC as well as recurrence after surgical resection of the primary tumor [21,22]. This evidence paved the way for the use of immune checkpoints in HCC, and many studies have shown promising results. Table 1 shows a summary of prospective studies with ICIs.

### 2.1. Nivolumab

In the phase III CheckMate 459 trial, nivolumab, a monoclonal antibody against PD-1, was compared to sorafenib as first-line treatment in 743 advanced HCC patients [23]. A preliminary report demonstrated that treatment with nivolumab at the dose of 240 mg intravenously (i.v.) every 2 weeks was associated with a two-fold higher objective response rate (ORR) (15% versus 7%) with 4% being complete responses (CR). Surprisingly, statistically significant results were not observed for either progression-free survival (PFS) (median: 3.7 vs. 3.8 months; HR: 0,93) or OS (median: 16.4 vs. 14.7; HR: 0.85, *p* = 0.0752). Grade 3 or higher treatment-related adverse events (TRAEs) were reported less often in nivolumab-treated patients (22% versus 49%), and the experimental arm was also less likely to discontinue therapy due to side effects (4% versus 8%).

Nivolumab was also evaluated as a second-line treatment in the multi-cohort CheckMate 040 trial for patients who showed intolerance or disease progression following treatment with sorafenib [24]. The trial predominantly included patients who were assigned Child–Pugh A score with or without viral etiology. Among the 212 patients who received nivolumab with a dose of 3 mg/kg every 2 weeks in the dose expansion phase, the ORR was 20% (42 patients) with three patients showing CRs. The median duration of response (DOR) was 9.9 months (95% CI: 8.3−not reached [NR] months), the median time to progression was 4.1 months (95% CI: 3.7−5.5 months), and the 9-month OS rate was 74% (95% CI: 67–79%). The drug was well tolerated with grade 3 and 4 TRAEs occurring in 19% of the patients included in the dose expansion cohort. Data of the 49 patients with Child–Pugh B tumors enrolled in these trials were also presented [25]. After 7.4 months of follow-up period, treatment with nivolumab resulted in a disease control rate of 55% and an ORR of 10% for such patients with poor prognosis. The median OS of the entire cohort was 7.6 months and was improved in sorafenib-naive patients (9.8 months) compared to that in patients with prior sorafenib exposure (7.3 months). These results led to a breakthrough approval of nivolumab by the United States Food and Drug Administration (FDA) on 22 September 2017, for the treatment of HCC in patients who are intolerant to sorafenib or demonstrated disease progression even with a sorafenib treatment regimen.

### 2.2. Pembrolizumab

In the phase II trial KEYNOTE-224, the effects of pembrolizumab were evaluated. Pembrolizumab was administered at a dose of 200 mg every 3 weeks to patients with advanced HCC (94% had Child–Pugh class A) who were previously treated with sorafenib [26]. Among the 104 patients, complete and partial responses were observed in one (1%) and 17 (16%) patients, respectively. In addition, stable disease (SD) was observed in 46 (44%) patients. The median OS was 12.9 months (95% CI: 9.7−15.5 months) and the median PFS was 4.9 months (95% CI: 3.4−7.2 months). Due to these encouraging results, the drug was also approved by the FDA in November 2018.

The placebo-controlled randomized phase III trial KEYNOTE-240 involved 413 patients; 278 patients received pembrolizumab (200 mg i.v. every 3 weeks) and 135 patients received placebo. After a median follow-up period of 13.8 months, patients who were treated with pembrolizumab demonstrated a numerical improvement in median OS (13.9 vs. 10.3 months, HR: 0.78; *p* = 0.0238) and median PFS (3.0 vs. 2.8 months, HR: 0.775; *p* = 0.0186), although the pre-specified co-primary endpoint of the statistically significant difference was not reached for both endpoints. However, the ORR of 16.9% (95% CI: 12.7−21.8%) for pembrolizumab versus 2.2% (95% CI: 0.5−6.4%) for placebo (nominal one-sided *p* = 0.00001) demonstrated a statistically significant improvement that was consistent with the results of KEYNOTE-224 [27].

### 2.3. Durvalumab

Durvalumab, an anti-PD-L1 monoclonal antibody, was evaluated in patients with HCC in a multi-cohort phase I/II trial. The trial enrolled 40 patients with HCC and Child–Pugh A to receive durvalumab at a dose of 10 mg/kg every 2 weeks for up to 12 months or until disease progression. The ORR was 10.3% (95% CI: 2.9–24.2%) and median OS was 13.2 months (95% CI: 6.3–21.1 months) in the entire population. Results were promising in the subgroup of patients with hepatitis C virus (HCV) (*n* = 8). In this subgroup, the ORR was 25.0% (95% CI: 3.2–65.1%) with a median OS of 19.3 months (95% CI: 9.5−23.0 months) [28].

A multi-arm study is evaluating durvalumab and tremelimumab alone or in combination for patients with advanced HCC that progressed on or were intolerant to sorafenib. The durvalumab monotherapy arm enrolled 104 patients to receive 1.500 mg of durvalumab every 4 weeks. The median OS for these patients was 11.7 months (95% CI: 8.5−16.9) and the ORR was 9.6% (95% CI: 4.7−17.0%) with a median DOR of 14.8 months [29].

### 2.4. Tremelimumab

In the second-line treatment, tremelimumab (a monoclonal antibody targeting CTLA4) administered at a dose of 15 mg/kg i.v. every 90 days was first evaluated in a single-arm phase II multicenter clinical trial with a sample of twenty patients with advanced HCC and hepatitis C infection etiology [30]. Approximately 43% of patients had Child–Pugh B disease. The study reported partial responses (PR) in 3 patients (17.6%), while 10 patients (58.8%) demonstrated an SD with a median PFS of 6.48 months (95% CI: 3.95−9.14 months).

The multi-arm trial evaluating tremelimumab and durvalumab for patients with advanced HCC also reported results for the tremelimumab monotherapy arm. Sixty-nine patients received 750 mg of tremelimumab every 4 weeks for seven doses followed by the same dose every four weeks thereafter. The median OS was 17.1 months (95% CI: 10.9−NR months), the ORR was 7.2% (95% CI: 2.4−16.1%) and the median DOR was 24 months [29].

### 2.5. Tislelizumab

Tislelizumab is a humanized anti-PD-1 antibody that was engineered to minimize binding to FcγR on macrophages, and therefore, to avoid antibody-dependent phagocytosis. Its effects were evaluated in a phase I trial with expansion cohorts. Patients with sorafenib-refractory HCC were involved in one of the expansion cohorts, who were subjected to treatment with tislelizumab at a dose of 5 mg/kg administered every 3 weeks. Data presented in 2017 demonstrating effects in 11 enrolled patients showed that the drug was safe and showed promising effects. Among the 10 evaluable patients, 1 patient demonstrated a PR and 6 showed SD as the best response [31].

### 2.6. Camrelizumab

Camrelizumab, a fully humanized anti-PD-1 monoclonal antibody (also known as SHR-1210), was tested in an open-label Chinese study with patients who were intolerant to first-line treatment or demonstrated progressive disease. Two hundred and seventeen eligible patients were randomly assigned to receive camrelizumab at a dose of 3 mg/kg intravenously every 2 or 3 weeks. With a median follow-up period of 125 months, the ORR for the entire population was 14.7% (95% CI: 10.3−20.2%). The median OS for all patients was 13.8 months (95% CI: 11.5−16.6 months) and median PFS was 2.1 months (95% CI: 2.0−3.2 months). Treatment was well-tolerated, and increased aspartate aminotransferase (5%) and neutropenia (3%) were the most commonly reported grade 3 or higher TRAEs [32].

### 2.7. Cemiplimab

Cemiplimab is another anti-PD-1 monoclonal antibody that is being tested as a treatment option in patients with advanced HCC. Results from a phase I trial with an expansion cohort were presented in 2018. The study involved patients with advanced HCC that had progressed after the administration of first-line treatment. Twenty-six patients were enrolled in the study. The median duration of follow-up was 7.2 months. PR was observed in five patients (ORR of 19.2%), and 14 patients (53.8%) showed SD. The median PFS was 3.7 months (95% CI: 2.3−9.1 months) [33].

### 2.8. Nivolumab + Ipilimumab

Patients with chronic HCV infection who receive treatment with ICIs that block either PD-L1/PD-1 or CTLA4 demonstrate an increased expression of circulating CD8+ T cells, but the activity of intrahepatic CD8+ T cells is not altered. However, the combination of anti-CTLA4 and anti-PD-L1/PD-1 inhibitors showed a synergistic effect in preclinical trials that reversed the refractoriness of intrahepatic CD8+ T cells [34]. The combination of ipilimumab (a monoclonal antibody against CTLA4) and nivolumab in clinical trials demonstrated the aforementioned potential synergistic effect. Patients included in the ipilimumab + nivolumab treatment cohort of the randomized CheckMate 040 trial were assigned to three treatment arms: (A) four doses of 1 mg/kg nivolumab + 3 mg/kg ipilimumab administered every 3 weeks followed by the administration of nivolumab at a fixed dose of 240 mg every 2 weeks, (B) four doses of 3 mg/kg nivolumab + 1 mg/kg ipilimumab administered every 3 weeks followed by the administration of nivolumab at a fixed dose of 240 mg every 2 weeks, or (C) administration of 3 mg/kg nivolumab every 2 weeks + 1 mg/kg ipilimumab every 6 weeks. The randomized study included 148 patients previously treated with sorafenib. The ORR was 32% (95% CI: 20%−47%) in arm A, 27% (95% CI: 15−41%) in arm B, and 29% (95% CI: 17−43%) in arm C. The median DOR was not reached (95% CI: 8.3−33.7 months) in patients involved in arm A, was 15.2 months (95% CI: 4.2−29.9 months) in patients involved in arm B and 21.7 months (95% CI: 2.8−32.7 months) in arm C. The median OS was 22.8 months (95% CI: 9.4−NR months) in arm A vs. 12.5 months (95% CI: 7.6−16.4 months) in arm B and 12.7 months (95% CI: 7.4−33.0 months) in arm C. This combination was well tolerated among the patients. Overall, patients included in arm A demonstrated more any-grade TRAEs than those in arms B and C (94% in arm A, 71% in arm B, and 79% in arm C) [35]. Based on this trial, on 10 March 2020, the FDA granted accelerated approval for the combination of ipilimumab + nivolumab as the treatment option for patients with HCC that progressed after treatment with sorafenib.

### 2.9. Tremelimumab + Durvalumab

Tremelimumab is being evaluated in combination with liver-directed therapies and durvalumab. A phase I/II trial is analyzing the effects of the combination of 20 mg/kg durvalumab and 1 mg/kg tremelimumab administered every 4 weeks for 4 cycles followed by durvalumab 20 mg/kg monotherapy every 4 weeks in patients with unresectable HCC with or without HBV or HCV. The first results observed in 40 patients (20 patients had HBV or HCV infection and 20 were uninfected) were presented in 2017. The majority of the patients (70%) had received prior systemic treatment, and 93% of patients were classified as Child–Pugh A. The confirmed ORR was 15% for all patients and 30% for uninfected patients. At the time of the interim analysis, no confirmed responses were observed among patients with HBV and HCV [36].

This combination is also being evaluated in another trial with different regimens. Patients with advanced HCC that were ICI-naïve and had progressed on, were intolerant to, or refused sorafenib were randomized to two combination regimens: 1) 300 mg of tremelimumab for one dose associated with 1.500 mg of durvalumab that continued every 4 weeks (*n* = 75); 2) 75 mg of tremelimumab every 4 weeks for 4 doses associated with 1.500 mg of durvalumab that continued every 4 weeks (*n* = 84). The median OS for arm 1 was 18.7 months (95% CI: 10.8−NR months) and for arm 2, it was 11.3 months (95% CI: 8.4−14.6 months). The ORR was 22.7% for arm 1 (95% CI: 13.8−33.8%) and 9.5% for arm 2 (95% CI: 4.2−17.9%). The median DOR was not reached in arm 1 and was 13.2 months for arm 2 [29].

## 3. Targeted Therapies

HCC has a complex molecular pathogenesis involving several signaling cascades such as epidermal growth factor receptor (EGFR), vascular endothelial growth factor receptor (VEGFR), platelet-derived growth factor receptor (PDGFR), hepatocyte growth factor (HGF/MET), and mechanistic target of rapamycin (mTOR) among others [37]. The growing knowledge of such molecular alterations harboring potential therapeutic targets gave us the rationale to clinically test tyrosine kinase inhibitors (TKI) targeting one or several of these pathways. The survival benefit of sorafenib [12] was the first successful targeted therapy approved in HCC and paved the way for the development of other targeted therapies. Table 2 is summarizing the results of the most relevant trials involving targeted therapies in advanced HCC patients.

### 3.1. Lenvatinib

Lenvatinib is a potent multi-TKI inhibitor that targets VEGF receptors (VEGFR1-3) and other pro-oncogenic tyrosine kinases, including fibroblast growth factor receptors (FGFR1-4), PDGFRα, KIT, and rearranged during transfection (RET) tyrosine kinases [38]. Recently, the use of levantinib as a first-line treatment for advanced HCC was approved based on the results of the phase III non-inferiority REFLECT trial, which compared lenvatinib (12 mg once daily for body weight ≥ 60 kg, 8 mg daily for <60 kg) versus sorafenib (400 mg twice daily for all patients). A total of 954 patients with unresectable HCC and no prior systemic therapy (99%Child–Pugh class A) were included in the trial. Patients with involvement of >50% of the liver or invasion of the main portal vein or biliary tree were excluded. Patients were randomly assigned to treatment with lenvatinib (*n* = 478) or sorafenib (*n* = 478). The median OS was 13.6 months for patients treated with levantinib and 12.3 months for patients treated with sorafenib (HR 0.92; 95% CI: 0.79−1.06 months). Median PFS was 7.4 months for patients treated with lenvatinib vs. 3.7 months for patients treated with sorafenib (HR: 0.66; *p* < 0.0001), and the ORR was 24.1 vs. 9.2% for lenvatinib and sorafenib treatments, respectively (OR 3.13; *p* < 0.0001). Grade 3 or higher TRAEs occurred in 57% of patients in the lenvatinib arm and 49% of patients in the sorafenib arm. The most common grade 3 or higher TRAEs in the lenvatinib arm were hypertension (23%) and decreased weight (8%) [39].

### 3.2. Donafenib

Donafenib is a multikinase inhibitor that targets Raf kinase and various receptor tyrosine kinases. This mechanism inhibits cell proliferation in Raf-expressing tumor cells. The efficacy of donafenib has been demonstrated according to an open-label randomized multicenter phase II/III trial with 668 patients with unresectable or metastatic HCC, Child–Pugh liver function score ≤ 7, and no prior systemic therapy. Patients were randomized to receive oral donafenib (0.2 g) or sorafenib (0.4 g) twice daily until intolerable toxicity or disease progression. The primary endpoint was OS. Donafenib showed potential benefits and significantly improved OS compared to sorafenib (12.1 vs. 10.3 months, HR 0.831; *p* = 0.0363). No significant differences were observed in median PFS (3.7 vs. 3.6 months for donafenib and sorafenib, respectively; *p* = 0.2824), ORR (4.6% vs. 2.7% for donafenib and sorafenib, respectively; *p* = 0.2448), and disease control rate (30.8% vs. 28.7% for donafenib and sorafenib, respectively; *p* = 0.5532). Grade 3 or higher TRAEs were reported in 57.4% and 67.5% of patients (*p* = 0.0082), respectively. Common adverse events reported in patients who received donafenib included skin reaction in hands and feet, increased aspartate aminotransferase levels, increased blood bilirubin levels, decreased platelet count, and diarrhea [40].

### 3.3. Cabozantinib

Cabozantinib is a potent inhibitor of several receptor tyrosine kinases, including HGF/c-MET, VEGFR-1, VEGFR-2, and VEGFR-3. The efficacy of cabozantinib in patients with previously treated advanced HCC was shown in phase III CELESTIAL trial. A total of 707 patients were enrolled with advanced and progressive HCC and no worse than Child–Pugh A cirrhosis. Patients were randomly assigned to cabozantinib (60 mg once daily) or placebo treatment groups. Eligible patients had received previous treatment with sorafenib, had disease progression after at least one systemic treatment for HCC, and may have received up to two previous systemic regimens for advanced HCC. The primary endpoint was OS. Treatment with cabozantinib prolonged median OS (10.2 vs. 8.0 months, HR 0.76; *p* = 0.005). Median PFS was 5.2 months in patients who were treated with cabozantinib and 1.9 months in patients treated with placebo (HR 0.44; *p* < 0.001). The ORR was 4% for cabozantinib and less than 1% for placebo treatment (*p* = 0.009). Grade 3 or 4 TRAEs were reported in 68% of patients included in the cabozantinib treatment group and 36% of the patients included in the placebo group. Palmar–plantar erythrodysesthesia, hypertension, fatigue, increased aspartate aminotransferase levels, and diarrhea were the most commonly reported high-grade TRAEs with cabozantinib treatment [41].

### 3.4. Regorafenib

Regorafenib is an orally active inhibitor of angiogenic (including VEGFR-1, VEGFR-2, and VEGFR-3), stromal, and oncogenic receptor tyrosine kinases. It is structurally similar to sorafenib and targets a variety of kinases implicated in angiogenic and tumor growth-promoting pathways. The advantage of regorafenib treatment in patients showing disease progression after first-line treatment with sorafenib was demonstrated in the RESORCE trial. A total of 573 patients were enrolled in this study. Patients had an Eastern Cooperative Oncology Group (ECOG) performance status of 0 to 1, Child–Pugh A score, and were randomly assigned to oral regorafenib 160 mg or placebo once daily during weeks 1−3 of each 4-week cycle. The primary endpoint was OS. Regorafenib was associated with significant prolongation of median OS (10.6 versus 7.8 months, HR 0.63; *p* < 0.0001). Median PFS was 3.1 months for patients treated with regorafenib and 1.5 months for patients treated with placebo (HR 0.46; *p* < 0.0001), and ORR was 11 vs. 4% for regorafenib and placebo, respectively (*p* = 0.0047). Grade 3 or 4 TRAEs were reported in 67% of patients in the regorafenib arm and 39% in the placebo arm. Most common grade 3 or higher TRAEs in regorafenib arm were hypertension (15%), hand–foot skin reaction (13%), and increased AST [42].

### 3.5. Ramucirumab

Ramucirumab is a recombinant monoclonal antibody belonging to immunoglobulin G subclass 1 (IgG1) that binds to VEGFR-2 and blocks receptor activation. In the REACH study, 565 patients who had failed previous treatment with sorafenib and continuously demonstrated Child–Pugh A score were randomly assigned to ramucirumab (8 mg/kg) or placebo every 2 weeks. The primary endpoint was OS. In the intention-to-treat population, the use of ramucirumab did not result in a significant gain in OS (9.2 vs. 7.6 months for ramucirumab and placebo, respectively; HR 0.87; *p* = 0.14). Median PFS was 2.8 months in the ramucirumab group versus 2.1 months in the placebo group (HR 0.63; *p* < 0.0001). The ORR was 7% for ramucirumab and < 1% for placebo (*p* < 0.0001). In this study, the analysis of a pre-specified subgroup of patients with alpha-fetoprotein levels (AFP) > 400 ng/mL indicated a potential benefit in OS upon treatment with ramucirumab (7.8 vs. 4.2 months; HR 0.67; *p* = 0.0059) [43]. A follow-up phase III trial (REACH-2) randomly assigned 292 HCC patients, Child–Pugh class A liver disease, who demonstrated disease progression after first-line sorafenib treatment and serum AFP ≥ 400 ng/mL. The patients received 8 mg/kg intravenous ramucirumab every 2 weeks or placebo treatment groups. The primary endpoint was OS. Ramucirumab was associated with a significantly better median OS, which was reported as 8.5 vs. 7.3 months in patients who received ramucirumab and placebo treatments, respectively (HR 0.71; *p* = 0.019). Median PFS was 2.8 months for ramucirumab vs. 1.6 months for placebo (HR 0.452; *p* < 0.0001). The ORR was 5 and 1% for ramucirumab and placebo, respectively (*p* = 0.1697). The most frequently reported TRAEs in ramucirumab arm were fatigue (27%), peripheral edema (25%) and decreased appetite (23%) [44]. Based on this trial, ramucirumab was approved in May 2019 for second-line treatment of HCC in patients with an AFP level ≥ 400 ng/mL.

### 3.6. Apatinib

Apatinib is an orally active VEGFR-2 inhibitor approved for second-line treatment of advanced gastric cancer in China. The efficacy of second-line treatment for advanced HCC after the failure of sorafenib and oxaliplatin-based chemotherapy was shown in a phase III randomized placebo-controlled trial of 393 patients with Child–Pugh A or B (≤7) cirrhosis. The patients received 750 mg apatinib orally once daily or placebo. The primary endpoint was OS. Apatinib significantly prolonged median OS (8.7 months in the apatinib arm versus 6.8 months in the placebo arm, HR 0.785; *p* = 0.0476) and median PFS (4.5 months with apatinib vs. 1.9 months with placebo; HR 0.471; p˂ 0.0001). The ORR was 10.7% (95% CI: 7.2−15.1%) in the apatinib group vs. 1.5% (95% CI: 0.2−5.4%) in the placebo group. TRAEs were reported in 97.3% of patients who received apatinib, with the most common AEs of grade 3 and 4 including hypertension, hand–foot syndrome, decreased platelet count, and decreased neutrophil count [45].

## 4. Combination of Immunotherapy and Targeted Therapies

The angiogenic pathway has immunosuppressive effects on the tumor microenvironment (TME) [46]. The VEGF pathway can negatively affect effector T cells and antigen-presenting cells and enhance the activity of immune suppressive cells such as regulatory T cells (Treg) and myeloid-derived suppressor cells (MDSCs) [47]. Anti-angiogenic targeted therapies can induce immunogenic alterations in the TME that can synergize with the effects of ICIs. Preclinical studies have demonstrated that anti-angiogenic TKIs can reduce the percentage of immunosuppressive Treg cells and MDSCs and increase T cell infiltration [48,49]. These findings were also confirmed in clinical studies [50] and successful combinations have been reported in multiple cancer types, such as clear cell renal cell carcinoma (ccRCC) [51,52,53], urothelial carcinoma [54], and endometrial carcinoma [55]. Both ICIs and anti-angiogenic targeted therapies are active in patients with metastatic HCC, making the combination of these two classes of treatment very attractive for these patients. Recent clinical data have confirmed the advantages. Table 3 summarizes the studies combining ICI and targeted therapy.

### 4.1. Atezolizumab + Bevacizumab

The combination of atezolizumab and bevacizumab showed effective results in patients with metastatic ccRCC [52]. This combination was also evaluated in patients with HCC. The phase IB study GO30140 evaluated atezolizumab alone or in combination with bevacizumab in patients with advanced HCC and no previous systemic treatment. Arm F randomized 119 patients 1:1 to 1200 mg i.v. of atezolizumab alone or in combination with 15 mg/kg i.v. of bevacizumab every 3 weeks. Arm A evaluated the combination of atezolizumab and bevacizumab (same dose as arm F) in 104 patients. The results showed a statistically significant benefit in median PFS, which was the primary endpoint for arm F, in favor of the combination over atezolizumab monotherapy (5.6 vs. 3.4 months; HR: 0.55; *p* = 0.0108). Arm A showed an ORR of 36% (primary endpoint) and a median PFS of 7.3 months (95% CI: 5.4−9.9 months) for patients that received the combination of atezolizumab and bevacizumab. Grade 3 or 4 TRAEs occurred in 20% of patients treated with the combination in arm F and 39% in arm A [56].

The phase III trial IMBRAVE 150 randomly assigned 501 previously untreated patients with advanced unresectable HCC and Child–Pugh A 2:1 to atezolizumab (1200 mg i.v. every three weeks) plus bevacizumab (15 mg/kg i.v. every three weeks) or sorafenib (400 mg twice daily) treatment groups [57]. The median OS was not reached for the patients included in the atezolizumab/bevacizumab arms and was 13.2 months among the patients included in the sorafenib arm (HR: 0.58, *p* < 0.001). Median PFS was 6.8 vs. 4.3 months for atezolizumab/bevacizumab and sorafenib treatment groups, respectively (HR: 0.59; *p* < 0.001). The ORR was 27.3% (95% CI: 22.5−32.5%) in patients treated with atezolizumab/bevacizumab patients vs. 11.9% (95% CI: 7.4−18.0%) in patients treated with sorafenib, based on the independent assessment performed in accordance with RECIST 1.1 (*p* < 0.001). In addition to the aforementioned results, the same trial showed that atezolizumab/bevacizumab was significantly associated with improved physical functioning (13.1 versus 4.9 months), role functioning (9.1 versus 3.6 months), and with longer delays in the median time to deterioration of quality of life (11.2 versus 3.2 months). Grade 3 or 4 TRAEs were reported in a similar percentage of patients in both groups (56.6% and 55.1% for atezolizumab/bevacizumab and sorafenib, respectively) [57]. Given the results of this trial, the FDA approved this treatment regimen in May 2020 for patients with unresectable or metastatic HCC who have not received prior systemic therapy.

### 4.2. Pembrolizumab + Lenvatinib

Pembrolizumab in combination with lenvatinib demonstrated effective results in patients with recurrent endometrial cancer [55], patients with ccRCC previously treated with ICIs [58], and patients with advanced gastric cancer [59]. Recently, a phase IB study aiming to evaluate the effects of lenvatinib (at a dose of 12 mg orally daily if body weight > 60 kg and 8 mg if < 60 kg) plus pembrolizumab (200 mg i.v.; every 3 weeks) in a single arm comprising 100 patients showed promising antitumor activity in unresectable or metastatic HCC. The ORR analyzed by independent imaging review (IIR) was 46% per modified Response Evaluation Criteria in Solid Tumors (mRECIST) with 11% of CR. Per RECIST version 1.1. (v1.1), the ORR was 36% with 1% CR. Median PFS according to the IIR was 9.3 months (95% CI: 5.6−9.7 months) per mRECIST and 8.6 months (95% CI: 7.1−9.7 months) per RECIST v1.1. Median OS was 22.0 months (95% CI: 20.4−NR months) per mRECIST criteria. Moreover, responses were considered durable with median DOR of 8.6 months (95% CI: 6.9–NR months) per mRECIST and 12.6 months (95% CI: 6.9−NR months) per RECIST v1.1, as evaluated by IIR. Grade 3 or 4 TRAEs were reported in 67% of patients, with hypertension being the most common adverse effect (17%), followed by AST increase (11%) and diarrhea (5%) [60].

### 4.3. Cabozantinib + Nivolumab and Cabozantinib + Nivolumab + Ipilimumab

Cabozantinib in combination with nivolumab with or without ipilimumab was evaluated in patients with metastatic urothelial carcinoma and other genitourinary tumors in a phase I trial. The treatment demonstrated manageable toxicity profiles and promising results [54]. These regimens are also being evaluated in patients with HCC. The CheckMate 040 study evaluated patients with advanced HCC that were treatment-naïve or had previously received sorafenib. Patients were randomly assigned to two treatment groups: The first group received nivolumab treatment at a dose of 240 mg i.v. every 2 weeks along with daily oral administration of 40 mg cabozantinib. The second group involved treatment with 3 mg/kg i.v. nivolumab every 2 weeks along with 40 mg cabozantinib and 1 mg/kg i.v. ipilimumab every 6 weeks. A total of 71 patients were enrolled, 36 for the doublet and 35 for the triplet regimen. The ORR was 17% and 26% for the doublet and triplet regimens, respectively. The median PFS was 5.5 months in patients treated with cabozantinib + nivolumab and 6.8 months in patients treated with cabozantinib, nivolumab + ipilimumab. The median OS was not reached in patients included in either arm. As expected, patients subjected to treatment with the triplet regimen demonstrated more grade 3 or 4 TRAEs (71% versus 42% for triplet versus doublet) [61].

### 4.4. Avelumab + Axitinib

Avelumab in combination with axitinib has shown promising results in patients with ccRCC with significant improvements in PFS [51]. This combination is also being evaluated in the phase IB VEGF liver 100 trial. This trial enrolled advanced HCC patients with an ECOG performance status of 0 or 1 and Child–Pugh class A who were subjected to treatment with 10 mg/kg i.v. avelumab every 2 weeks in combination with axitinib 5 mg that was administered twice daily via the oral route. Interim results presented in 2019 showed that the most common grade 3 TRAEs were hypertension (50.0%) and hand–foot syndrome (22.7%). The ORR per RECIST was 13.6% (95% CI: 2.9−34.9%), per mRECIST—31.8% (95% CI: 13.9−54.9%). Median PFS was 5.5 months per RECIST (95% CI: 1.9−7.3 months) and 3.8 months per mRECIST (95% CI: 1.9−7.3 months) [62].

### 4.5. Camrelizumab + Apatinib

The anti-PD-1 antibody camrelizumab (SHR-1210) is being evaluated in combination with apatinib in patients with advanced HCC and gastric cancer (GC) or esophagogastric junction cancer (EGJC). The results of a phase I trial with a dose escalation and expansion cohorts were recently reported [63]. A total of 43 patients were enrolled in the study (18 with HCC and 25 with GC/EGJC). Fifteen patients (83.3%) demonstrated disease progression or were intolerant to sorafenib and 13 had Child–Pugh A score. The recommended phase II dose for apatinib was 250 mg and the dose of camrelizumab was 200 mg i.v. every 2 weeks. The most common grade 3 or higher TRAEs were hypertension (15.2%) and elevated aspartate aminotransferase levels (AST; 15.2%). Among patients with HCC, 16 were considered evaluable. Among them, eight patients demonstrated PR (50%) and 7 patients showed SD (43.7%). The median PFS of patients with HCC was 5.8 months (95% CI: 2.6−NR months), and the median OS was not reached.

## 5. Biomarkers

### 5.1. Biomarkers for Targeted Therapy

Multiple genes could be affected during the carcinogenesis of HCC, including the telomerase reverse transcriptase (TERT) promoter, beta-1 catenin (CTNNB1), TP53, axis inhibitor-1 (AXIN1), AT-rich interaction domain-containing protein 1A (ARID1A), nuclear factor erythroid 2-like 2 (NFE2L2), ARID2, tuberous sclerosis 1 (TSC1), TSC2, and ribosomal protein S6 kinase 90-kD 3 (RPS6KA3) [64].

Unfortunately, only a minority of the genomic alterations observed in HCC are targetable. However, none of these alterations are currently used in clinical practice to guide treatment. Deeper sequencing of the HCC genomic profiling can identify new candidate biomarkers. For instance, after the whole genome analysis of responders and non-responders, Lee et al. [65] observed that non-synonymous single nucleotide variations in solute carrier family 15 (H+/peptide transporter) member 2 (SLC15A2) were associated with better outcomes in patients treated with sorafenib.

### 5.2. Biomarkers for Immunotherapy

#### 5.2.1. PD-L1 Expression

PD-1/PD-L1 checkpoint inhibitors are antibodies against these membrane receptors (PD-1 and PD-L1) involved in controlling T cell activation. Therefore, PD-L1 expression, typically assessed by immunohistochemistry, has emerged as an obvious predictor of response to checkpoint inhibitors.

Overexpression of PD-L1 has been detected in the microenvironment of HCC tumors [66], and it might be associated with worse prognosis [67]. Although it is not currently used in clinical practice for tailoring therapy in HCC, studies have suggested a potential predictive value. For instance, Sangro et al. analyzed tumor samples from the CheckMate 040 trial and showed that PD-L1 ≥ 1% was associated with better OS (28.1 vs. 16.6 months, *p* = 0.03) when comparing with PD-L1 < 1% [68].

However, the assessment of PD-L1 expression is challenged by the biological heterogeneity of the sampled tissue and clonal diversity of the antibodies used for its detection. In addition, different methodologies are used for scoring PD-L1 expression in tumor and infiltrating cells. Not surprisingly, its predictive ability varies largely across different types of malignancies and even in the same type of cancer, such as HCC. The analytical heterogeneity of PD-L1 assays has led to poor performance of this biomarker. For instance, in the Keynote-224 trial, which evaluated PD-L1 expression using the 22c3 PharmDx assay, the combined tumoral and stromal PD-L1 expression was associated with better outcomes along with the use of pembrolizumab, whereas PD-L1 expression in tumor cells did not demonstrate such results [26]. The current FDA-approved first-line therapy for advanced HCC involving atezolizumab (an anti-PD-L1 antibody) + bevacizumab (an anti-VEGF antibody) regimen does not require the assessment of PD-L1 expression for its use.

#### 5.2.2. Tumor Mutational Burden (TMB)

TMB is defined as the total number of non-synonymous mutations in the tumor exome [69], typically measured as mutations per megabase (mut/Mb). The rationale for its use as a biomarker for immunotherapy is that the higher the levels of somatic mutations, the higher the expression of neoantigens that are not subjected to immune tolerance. In other words, it is believed that tumors harboring > 10 mut/Mb are enriched in tumor neoantigens capable of recognition by T cells and, therefore, more susceptible to immune system action once mechanisms of tumor evasion from the immune response are canceled by checkpoint inhibitors. This looks like a continuous variable with higher numbers of TMB meaning higher response rates. Unfortunately, HCC has a low median TMB (5 Mut/Mb) [70]. Clinical conditions involving higher TMB, such as microsatellite instability, are very rare in HCC. Ang et al. analyzed more than 700 tumors, but only six tumors (0.8%) were TMB-high and, out of the 542 cases assessed for MSI, only one (0.2%) was MSI-high and TMB-high. They reported one patient (TMB: 15 mutations/Mb, MSI-low) with a sustained complete response to nivolumab lasting > 2 years [71]. In a study by Kawaoka, two patients (2.4%) had MSI-H tumors: one of them had a complete response to pembrolizumab for over 10 months, and the other was a non-responder [72].

Hence, the use of TMB in HCC is still under investigation. Based on this information and the fact that immunotherapy is now approved as the first line for HCC irrespective of the TMB, such analysis does not seem to be of use in this pathology nowadays.

#### 5.2.3. Intestinal Microbiota

The intestinal microbiome has been linked with response/resistance checkpoint inhibitors in different malignancies, and studies on HCC are emerging. Yi Zheng et al. analyzed fecal samples from 8 advanced HCC patients treated with immunotherapy after progression under sorafenib and showed that the enrichment of some species, including *Akkermansia* and *Ruminococcaceae*, may predict the response. The results also suggested that the intestinal microbiota profile may serve as a predictive clinical biomarker for evaluating checkpoint inhibitors. Also, by way of metagenomic sequencing of periodic fecal samples during anti-PD-1 immunotherapy, they showed an increase in *Proteobacteria* among non-responders, demonstrating that dynamic variation characteristics of the intestinal microbiome might be used for early prediction of benefits of immunotherapy [73]. Studies with larger patient cohorts are needed.

#### 5.2.4. WNT/β-Catenin Signaling

The Wnt/β-catenin pathway regulates liver homeostasis and zonation [74]. Activating mutations in β-catenin (CTNNB1), present in 15−33% of HCC patients [75], can lead to aberrant Wnt signaling activation. Recent studies have suggested an important role of upregulation of the β-catenin (CTNNB1) pathway with both immune evasion and resistance to anti-PD-1 therapy [76].

The relationship between β-catenin pathway upregulation and response to immunotherapy was demonstrated in a study by Harding et al., where 31 patients with advanced HCC were treated with immune checkpoint inhibitors. Those with activating alteration in WNT/β-catenin signaling had lower disease control rate (0% vs. 53%), shorter median PFS (2.0 vs. 7.4 months; HR, 9.2; 95% CI:2.9–28.8; *p* < 0.0001), and shorter median OS (9.1 vs. 15.2 months; HR, 2.6; 95% CI: 0.76–8.7; *p* = 0.11) [77].

## 6. Perspectives

Currently, phase III randomized clinical trials of nivolumab, atezolizumab, durvalumab, pembrolizumab, ipilimumab, tremelimumab, and tislelizumab in monotherapy or combination therapy are being conducted (Table 4). The RATIONALE-301 and HIMALAYA study are two ongoing trials. RATIONALE 301 is a phase III randomized open-label study that aims to evaluate the efficacy and safety of tislelizumab compared with sorafenib as the first-line treatment in adult patients with unresectable HCC [78]. The HIMALAYA study is a phase III trial that aims to analyze the effects of durvalumab in conjunction with tremelimumab compared to durvalumab monotherapy or sorafenib as first-line therapy for HCC (NCT03298451).

New ways to optimize the use of immunotherapy in HCC have been studied. Dysregulated TGF-β pathway, which exhibits many functions in the liver tissue, is likely to be involved in immune evasion by HCC tumor cells [79]. Two of the many proposed mechanisms involve the upregulation of PD-1 [80] and the promotion of the deposition of extracellular matrix proteins in the microenvironment by myofibroblast activation, which could lead to a decrease in T cell infiltration [81,82].

Galunisertib, a TGF-β receptor 1 kinase inhibitor, was evaluated recently in a phase II study. This trial enrolled 149 patients with advanced HCC, Child–Pugh A/B7, that progressed under or were ineligible to receive sorafenib. Patients were evaluated in two cohorts according to the baseline serum AFP value: one hundred and nine patients were enrolled in Part A [AFP ≥ 1.5 × upper limit of normality (ULN)] and 40 patients—in Part B (AFP < 1.5 × ULN). Patients received galunisertib 80 to 150 mg orally twice a day for 14 days on a 28-day cycle. The primary endpoint was time-to-tumor progression (TTP) and the effect of treatment on plasma levels of TGF-β and AFP. Median TTP was 2.7 months (95% CI: 1.5−2.9 months) in Part A and 4.2 months (95% CI: 1.7−5.5 months) in Part B. The median OS was 7.3 months (95% CI: 4.9−10.5 months) in Part A and 16.8 months (95% CI: 10.5−24.4 months) in Part B. Patients who had a response (decrease from baseline > 20%) on both AFP and TGF-β1 during therapy with galunisertib had a significantly prolonged median OS versus non-responders (21.5 vs. 6.8 months; *p* = 0.0015) [83].

Another phase II study tested galunisertib (80 to 150 mg orally twice a day for 14 days on a 28-day cycle) in combination with sorafenib (400 mg twice a day) in patients with advanced HCC who did not receive previous systemic therapy. The study enrolled 47 patients. The median TTP was 4.1 months (95% CI: 2.8−6.5 months) and the median OS was 18.8 months (95% CI: 14.8−24.8 months). These results compare favorably with historical results for this population with sorafenib alone [12], suggesting that inhibition of TGF-β signaling may have delayed tumor resistance. However, as there was no control arm and no assessment of peripheral regulatory T cell counts in this study, it cannot be concluded whether the combination of agents had a synergistic or additive effect in improving outcomes in HCC [84]. Finally, a study of galunisertib in combination with nivolumab in advanced refractory solid tumors and in recurrent or refractory NSCLC or HCC is ongoing (NCT02423343).

## 7. Conclusions

Immunotherapy and targeted molecular therapies have personalized the treatment of metastatic HCC and have led to undoubted improvements in patient care. ICIs have shown promising results in phase I and II clinical trials; however, response rates are still low (15% to 19.2%). Phase III trials have failed to show significant improvement in OS with monotherapies (Table 1). The combination of two ICIs (anti-PD-1/PD-L1 and anti-CTLA4) has shown promising results in early trials and may statistically improve clinical outcomes over monotherapy. The combination of ICI with targeted therapy may demonstrate synergistic effects. Indeed, for the first time, a combination (atezolizumab and bevacizumab) treatment regimen was able to challenge sorafenib as the first-line treatment. Moreover, other phase I and II studies have shown promising results with the combination of these two classes achieving response rates of nearly 50% and a superior median OS of 20 months (Table 3).

The introduction of several treatment options in the first- and second-line scenario with high response rates and potential for long-term disease control is new in the field and raises several questions. Here, we will briefly mention three of them that are the most relevant ones in our opinion. (1) We will probably have three treatment options in the first line with the same level of evidence. Therefore, clinical characteristics and biomarkers should be profoundly considered and analyzed while the first-line treatment is being selected: immunotherapy/bevacizumab, combination immunotherapy (ongoing trials), or donatenib therapy. (2) Since we now have appreciable response rates near 30% and treatments that can also provide long-term disease control, there is room for local treatment either for local control, to boost immunotherapy effect, or even with curative intention after tumor downstaging. Specialists will have to evolve to play with ablations, embolization, surgeries, and radiosurgeries in this new era. (3) Even more challenging and less evidence-based is the selection of the second and further lines of treatment. Regorafenib and cabozantinib clinical trials did not include patients previously treated with immunotherapies [41,42], that now comprise the standard first line. Further complicating this issue, some specialists also consider that sorafenib or lenvatinib could be used as the first-line tyrosine kinase inhibitor following immunotherapy-based regimens [85].

Finally, the impact of HCC on global health is expected to continue to rise. In the last 13 years, important advances in the treatment of HCC and increased understanding of tumorigenesis and progression of cancer have been fueling investigation of biologic therapies. Although there is an urgent need to implement genome-based therapies, understanding the predictors of response to identify agents is essential, especially in primary resistant tumors.

## Figures and Tables

**Table 1:** Results of selected studies testing immune checkpoint inhibitors in advanced HCC patients.

Study (Year)	Phase	*n*	Population	Drug	Median overall Survival	Median Progression-Free Survival	Objective Response Rate
CheckMate 459 (2019) [23]	III	743	Unresectable Child–Pugh A HCC naïve to systemic treatment	Nivolumab vs. sorafenib	16.4 mo for nivolumab vs. 14.7 mo for sorafenib (HR: 0.85; *p* = 0.0752)	3.7 mo for nivolumab vs. 3.8 mo for sorafenib	15% for nivolumab and 7% to sorafenib
CheckMate 040 (2017) [24]	I/II	262 (dose escalation: 48 and dose expansion: 216)	Advanced HCC with or without HBV or HCV, Child–Pugh A or B7 after sorafenib failure or intolerance	Nivolumab	Dose escalation phase: 15.0 mo Dose expansion phase: 9-mo OS rate: 74%	Dose escalation phase: 3.4 mo Dose expansion phase: 4.1 mo	Dose escalation phase: 15% Dose expansion phase: 20%
KEYNOTE-224 (2018) [26]	II	104	Advanced HCC, Child–Pugh A after sorafenib failure or intolerance	Pembrolizumab	12.9 mo	4.9 mo	17%
KEYNOTE-240 (2020) [27]	III	413	Advanced HCC, Child–Pugh A after sorafenib failure or intolerance	Pembrolizumab vs. placebo	13.9 mo for pembrolizumab vs. 10.3 mo for placebo group (HR: 0.78; *p* = 0.0238)	3.0 mo in the pembrolizumab group vs. 2.8 mo in the placebo group (HR: 0.775; *p* = 0.0186)	16.9% for pembrolizumab vs. 2.2% for placebo
Wainberg et al. (2017) [28]	I/II	40	Advanced HCC, Child–Pugh A after sorafenib failure or intolerance	Durvalumab	13.2 mo		10%
Kelley et al. (2020) [29]	II	104	Advanced HCC after sorafenib failure or intolerance	Durvalumab	11.7 mo		9.6%
Sangro et al. (2013) [30]	II	20	HCV-related advanced HCC, Child–Pugh A or B	Tremelimumab	8.2 mo	6.48 mo	17,6%
Kelley et al. (2020) [29]	II	159	Advanced HCC after sorafenib failure or intolerance to sorafenib	Tremelimumab	17.1 mo		7.2%
Qin et al. (2020) [32]	II	217	Advanced HCC, Child–Pugh A or B7 after sorafenib failure or intolerance to first-line systemic therapy	Camrelizumab	13.8 mo	2.1 mo	14.7%
He et al. (2018) [33]	Ib	26	Advanced HCC, Child–Pugh A after failure or intolerance to first-line systemic therapy	Cemiplimab		3.7 mo	19.2%
Checkmate 040 (2020) [35]	II	148	Advanced HCC patients, Child–Pugh A previously treated or intolerant to sorafenib	Nivolumab + ipilimumab	Arm A: 22.8 mo Arm B: 12.5 mo Arm C: 12.7 mo		Arm A: 32% Arm B: 27% Arm C: 29%
Kelley et al. (2017) [36]	I/II	40	Advanced HCC with or without HBV or HCV, Child–Pugh A or B; 70% were previously treated.	Durvalumab + tremelimumab			15% for all patients and 30% for uninfected patients
Kelley et al. (2020) [29]	II	159	Advanced HCC who progressed on, were intolerant to, or refused sorafenib	Durvalumab + tremelimumab	18.7 mo for tremelimumab 300 mg + durvalumab and 11.3 months for tremelimumab 75 mg + durvalumab		22.7% for tremelimumab 300 mg + durvalumab and 9.5% for tremelimumab 75 mg + durvalumab

**Abbreviations:** HCC: hepatocellular carcinoma; mo: months; HR: hazard ratio; HBV: hepatitis B virus; HCV: hepatitis C virus; vs.: versus; NR: not reached; BCLC: Barcelona Clinic Liver Cancer; OS: overall survival.

**Table 2:** Results of selected studies testing targeted therapies in HCC patients.

Study (Year)	Phase	*n*	Population	Drug	Median Overall Survival	Median Progression-Free Survival	Objective Response Rate
REFLECT trial (2018) [39]	III non-inferiority	954	Unresectable HCC and no prior systemic therapy (99% Child–Turcotte–Pugh class A)	Lenvatinib vs. sorafenib	13.6 mo for lenvatinib vs. 12.3 mo for sorafenib (HR: 0.92, 95% CI: 0.79−1.06)	7.4 mo for lenvatinib vs. 3.7 mo for sorafenib (HR: 0.66; *p* < 0.0001)	24.1% for lenvatinib vs. 9.2% for sorafenib (*p* < 0.0001)
Feng Bi et al. (2020) [40]	II/III	668	Unresectable or metastatic HCC, Child–Pugh liver function score ≤ 7, and no prior systemic therapy	Donafenib vs. sorafenib	12.1 mo for donafenib vs. 10.3 mo for sorafenib (HR: 0.831; *p* = 0.0363)	3.7 mo for donafenib vs. 3.6 mo for sorafenib (*p* = 0.2824)	4.6% for donafenib vs. 2.7% for sorafenib (*p* = 0.2448)
CELESTIAL trial (2018) [41]	III	707	Advanced and progressing HCC and not worse than Child–Pugh A	Cabozantinib vs. placebo	10.2 mo for cabozantinib vs. 8.0 mo for placebo (HR: 0.76; *p* = 0.005)	5.2 mo for cabozantinib vs. 1.9 mo for placebo (HR: 0.44; *p* < 0.001)	4% for cabozantinib vs. less than 1% for placebo (*p* = 0.009)
RESORCE trial (2017) [42]	III	573	Advanced HCC that progressed after first-line treatment with sorafenib, Child–Pugh A	Regorafenib vs. placebo	10.6 mo for regorafenib vs. 7.8 mo for placebo (HR: 0.63; *p* < 0.0001)	3.1 mo for regorafenib vs. 1.5 mo for placebo (HR: 0.46; *p* < 0.0001)	11% for regorafenib vs. 4% for placebo (*p* = 0.0047)
REACH trial (2015) [43]	III	565	Advanced HCC following first-line therapy with sorafenib and Child–Pugh A	Ramucirumab vs. placebo	9.2 mo for ramucirumab vs. 7.6 mo for placebo (HR: 0.87; *p* = 0.14).	2.8 mo for ramucirumab vs. 2.1 mo for placebo (HR 0.63; *p*<0.0001)	7% for ramucirumab vs. < 1% for placebo (*p*<0.0001)
REACH-2 trial (2019) [44]	III	292	Advanced HCC, Child–Pugh class A, and serum AFP ≥ 400 ng/mL in patients who had disease progression under first-line sorafenib	Ramucirumab vs. placebo	8.5 mo for ramucirumab vs. 7.3 mo for placebo (HR: 0.71; *p* = 0.0199	2.8 mo for ramucirumab vs. 1.6 mo for placebo (HR: 0.452; *p* < 0. 0001)	5% for ramucirumab vs. 1% for placebo (*p* = 0.1697)
Qiu Li et al. (2020) [45]	III	393	Advanced HCC after failure of sorafenib and oxaliplatin-based chemotherapy and Child–Pugh liver function class A or B ≤ 7 points	Apatinib vs. placebo	8.7 mo for apatinib vs. 6.8 mo for placebo (HR: 0.785; *p* = 0.0476)	4.5 mo for apatinib vs. 1.9 mo for placebo (HR: 0.471; *p* ˂ 0.0001)	10.7% for ramucirumab vs. 1.5% for placebo

**Abbreviations:** HCC: hepatocellular carcinoma; mo: months; HR: hazard ratio.

**Table 3:** Results of selected studies testing the combination of immune checkpoint inhibitors and targeted therapies in advanced HCC patients.

Study (Year)	Phase	*n*	Population	Drug	Median Overall Survival	Median Progression-Free Survival	Objective Response Rate
GO30140 (2019) [56]	Ib	Arm F: 119 Arm A: 104	Unresectable HCC, Child–Pugh A, and naïve to systemic treatment	Atezolizumab + bevacizumab		Arm F: 5.6 mo for atezolizumab + bevacizumab vs. 3.4 mo for atezolizumab alone (HR: 0.55; *p* = 0.0108) Arm A: 7.3 mo	Arm A: 36%
IMbrave 150 (2020) [57]	III	501	Unresectable HCC, Child–Pugh A, and naïve to systemic treatment	Atezolizumab + bevacizumab vs. sorafenib	NR for the atezolizumab/bevacizumab group vs. 13.2 mo for the sorafenib group (HR: 0.58, *p* < 0.001)	6.8 vs. 4.3 mo for the atezolizumab/bevacizumab group and the sorafenib group, respectively (HR: 0.59; *p* < 0.001)	27.3% for the atezolizumab/bevacizumab group and 11.9% for the sorafenib group
Finn et al. (2020) [60]	Ib	30	Advanced HCC BCLC B/C, Child–Pugh A, in the first-line setting	Pembrolizumab + levantinib	22.0 mo	9.3 mo	46%
CheckMate 040 (2020) [61]	I/II	71	Advanced HCC patients that were treatment-naïve or that received sorafenib previously	Arm 1: CaboNivo Arm 2: CaboNivoIpi	NR in both arms	Arm 1: 5.5 mo Arm 2: 6.8 mo	Arm 1: 17% Arm 2: 26%
VEGF Liver 100 (2019) [62]	Ib	22	Advanced HCC, Child–Pugh A in the first-line setting	Avelumab + axitinib		5.5 mo per RECIST	13.6%
Xu et al. (2019) [63]	I	18	HCC patients, Child–Pugh A, and previously treated with sorafenib	SHR-1210 + apatinib	NR	5.8 mo	50%

**Abbreviations:** HCC: hepatocellular carcinoma; mo: months; HR: hazard ratio; NR: not reached; BCLC: Barcelona Clinic Liver Cancer; CaboNivo: cabozantinibe + nivolumab; CaboNivoIpi: cabozantinibe + nivolumab + ipilimumab.

**Table 4:** Ongoing phase III trials investigating immune checkpoint inhibitors in patients with advanced HCC.

Intervention	Comparison	Study name	NCT Number Trial	Primary Endpoint	Setting
Durvalumab + tremelimumab or durvalumab alone	Sorafenib	HIMALAYA	NCT03298451	OS	Palliative, 1st-line
Pembrolizumab + lenvantinib	Levantinib	LEAP-002	NCT03713593	PFS/OS	Palliative, 1st-line
Atezolizumab + cabozantinib	Sorafenib	COSMIC-312	NCT03755791	PFS/OS	Curative adjuvant
Camrelizumab + apatinib	Sorafenib	-	NCT03764293	OS, PFS	Palliative, 1st-line
Nivolumab + ipilimumab	Sorafenib or lenvantinib	CheckMate 9DW	NCT04039607	OS	Palliative, 1st-line
Nivolumab + lenvantinib	Levantinib	-	NCT04044651	OS	Palliative, 1st-line
Sintilimab + IBI305 (anti-VEGF)	Sorafenib	ORIENT-32	NCT03794440	OS, ORR	Palliative, 1st-line
Tislelizumab	Sorafenib	RATIONALE-301	NCT03412773	OS	Palliative, 1st-line
Pembrolizumab	Placebo	KEYNOTE-394	NCT03062358	OS	Palliative, 2nd-line
Pembrolizumab	Best supportive care	KEYNOTE-240	NCT02702401	OS/PFS	Palliative, 2nd-line

**Abbreviations:** OS: overall survival; PFS: progression-free survival; ORR: objective response rate.
